# Effects of serum estrogen levels before frozen-thawed blastocyst transfer on pregnancy outcomes in hormone replacement cycles

**DOI:** 10.1038/s41598-023-27877-w

**Published:** 2023-01-21

**Authors:** Yi-ran Du, Ke Yang, Jie Liu

**Affiliations:** 1 Medical College, Wuhan University of Science and Technology, Wuhan, 430070 China; 2 Reproductive Center, Maternal and Child Health Hospital of Hubei Province, Tongji Medical College of Huazhong University of Science and Technology, No. 745, Wuluo Road, Hongshan District, Wuhan, 430070 China

**Keywords:** Diseases, Endocrinology

## Abstract

We investigated the effects of serum estrogen levels before frozen-thawed blastocyst transfer on pregnancy outcomes in hormone replacement cycles. Clinical data of 708 hormone replacement cycles with frozen-thawed blastocyst were retrospectively analyzed. According to quartile (P25) of serum estrogen levels on the endometrium transformation day, the 708 cycles were divided into group A_1_ (E2 < 157.5 pg/ml), group A_2_ (157.5 pg/ml ≤ E2 < 206.4 pg/ml), group A_3_ (206.4 pg/ml ≤ E2 < 302.3 pg/ml) and group A_4_ (E2 ≥ 302.3 pg/ml). According to quartile (P25) of serum estrogen levels on the frozen-thawed blastocyst transfer day, the 708 cycles were divided into group B_1_ (E2 < 147 pg/ml), group B_2_ (147 pg/ml ≤ E2 < 200.4 pg/ml), group B_3_ (200.4 pg/ml ≤ E2 < 323 pg/ml) and group B_4_ (E2 ≥ 323 pg/ml). According to different clinical outcomes, the 708 cycles were divided into clinical pregnant group and non-clinical pregnant group. The group A_4_ (E2 ≥ 302.3 pg/ml on the endometrium transformation day) was significantly lower than other groups in blastocyst implantation rate and multiple-pregnancy rate (*P* < 0.05). The days of taking progynova was significantly different among groups on both endometrium transformation day and frozen-thawed blastocyst transfer day (*P* < 0.05), but there were no statistical differences in the mean age, endometrial thickness and number of high-quality blastocysts transferred among groups (*P* > 0.05). The mean age was significantly younger and the number of high-quality blastocysts transferred was significantly higher in the clinical pregnant group than in the non-clinical pregnant group (*P* < 0.05), but endometrial thickness, days of taking progynova, progesterone level on the blastocyst transfer day, and E2 level were not significantly different between both groups (*P* > 0.05). Multivariate regression analysis indicated that age was an independent factor affecting clinical pregnancy (*P* < 0.05). Correlation analysis displayed that the serum estrogen levels did not affect clinical pregnancy (*P* > 0.05). The days of taking progynova and serum estrogen levels before frozen-thawed blastocyst transfer do not affect pregnancy outcomes in hormone replacement cycles.

## Introduction

With progress of embryo cryopreservation, frozen-thawed blastocyst transfer has become a major approach of assisted reproduction^1^. There has been a debate on the optimal endometrial preparation protocol^2,3^. Using hormone replacement therapy (HRT) to prepare the endometrium makes it possible to flexibly arrange the date of embryo transfer and reduce the frequency of follicle monitoring, so HRT is very popular clinically^1^. In recent years, scholars have carried out a series of studies to optimize HRT protocol for improving the pregnancy outcome of frozen-thawed embryo transfer (FET). It has been reported that the endometrial thickness of 8.7–14.5 mm may obtained the optimal live birth rate, and when the endometrial thickness is too thin or too thick the live birth rate will decrease in HRT-FET cycles^4^. Studies have indicated that the progesterone level on the embryo transfer day is an independent predictor for pregnancy rate and live birth rate in HRT cycles^5,6^. However, the optimal estrogen level for the pregnancy outcomes of HRT cycles is not clear. In natural cycles, with follicular growth, the estrogen level gradually increases until more than 200 pg/ml in late follicular phase, and the estrogen level of 200 pg/ml lasts for 50 h at least^7^. Nevertheless, it is still unclear whether the estrogen level is associated with the pregnancy outcomes in HRT cycles. It has been reported that the estrogen level on the endometrium transformation day is not related to pregnancy outcomes in HRT cycles^8–11^, serum estrogen level > 100 pg/ml is one of the prerequisites for endometrium transformation^12^, and cancellation of embryo transfer is recommended when the serum estrogen level is less than 75 pg/ml^13^. In this study, we collected clinical data of 708 HRT-FET cycles performed in our hospital from January 2019 to December 2020 and retrospectively analyzed the effects of serum estrogen levels before embryo transfer on pregnancy outcomes, providing a clinical basis for improving HRT-FET protocol.

## Materials and methods

All study methods were approved by the Ethics Committee of the Hubei Maternal and Child Health Hospital (2022IEC081), and were performed in accordance with relevant guidelines and regulations. All the subjects enrolled into the study gave written informed consent to participate.

### Subjects

HRT-FET cycles including in vitro fertilization (IVF) and intracytoplasmic sperm injection (ICSI) performed in our reproductive center from January 2019 to December 2020, were collected. Inclusion criteria were (1) age ≤ 40 years; (2) endometrial thickness ≥ 8 mm; and (3) two blastocysts transferred. Exclusion criteria included (1) uterine cavity lesions and uterine malformations; (2) uterine fibroids; (3) endometriosis; (4) hydrosalpinx; (5) chromosome abnormality; (6) a history of thrombosis; (7) contraindication for estrogen; and (8) embryos that underwent PGTA. A total of 708 cycles were in line with above inclusion and exclusion criteria.

### Endometrial preparation and grouping

If B-ultrasound showed that the uterus and ovaries were normal on the third day of menstrual onset or drug withdrawal bleeding, patients started taking 2 mg of progynova twice a day for 4 days, and then 3 mg of progynova twice a day for next 4 days. The B-ultrasound was subsequently performed to show the endometrial thickness, and then the dose and days of taking progynova were adjusted according to the endometrial thickness. When the endometrial thickness was more than 8 mm, the levels of serum estrogen and progesterone were determined. If the progesterone level is less than 1.0 pg/ml, progesterone was injected. Five days later, 2 blastocysts were transplanted in the uterus. Before blastocyst transfer, the endometrial thickness was measured and the levels of estrogen and progesterone were determined. According to quartile (P25) of serum estrogen levels on the endometrium transformation day, the 708 cycles were divided into group A_1_ (E2 < 157.5 pg/ml, 176 cycles), group A_2_ (157.5 pg/ml ≤ E2 < 206.4 pg/ml, 178 cycles), group A_3_ (206.4 pg/ml ≤ E2 < 302.3 pg/ml, 176 cycles) and group A_4_ (E2 ≥ 302.3 pg/ml, 178 cycles). According to quartile (P25) of serum estrogen levels on the frozen-thawed blastocyst transfer day, the 708 cycles were divided into group B_1_ (E2 < 147 pg/ml, 176 cycles), group B_2_ (147 pg/ml ≤ E2 < 200.4 pg/ml, 178 cycles), group B_3_ (200.4 pg/ml ≤ E2 < 323 pg/ml, 176 cycles) and group B_4_ (E2 ≥ 323 pg/ml, 178 cycles). Clinical characteristics including age, endometrial thickness on the endometrium transformation day, endometrial thickness on the blastocyst transfer day, days of taking progynova, progesterone level on the blastocyst transfer day and number of high-quality blastocysts transferred as well as pregnancy outcomes including clinical pregnancy rate, blastocyst implantation rate, multiple-pregnancy rate, abortion rate and live birth rate were compared between groups. According to different clinical outcomes, the 708 cycles were divided into clinical pregnant group (n = 520) and non-clinical pregnant group (n = 188). The estrogen levels on the endometrium transformation day and blastocyst transfer day were compared between the two groups. Multivariate regression analysis of clinical pregnancy and correlation analysis between clinical pregnancy and serum E2 level were performed.

### Blastocyst cryo-resuscitation

According to Gardner scoring system^14^, the blastocysts with above stage 3 of blastocyst cavity expansion, above grade B of inner cell mass and above grade C of trophectoderm underwent cryopreservation. Vitrification and thawing were used in transferred blastocysts. Expansion of blastocyst cavity indicated survival of thawed blastocysts. These blastocysts were thawed 2 h before blastocyst transfer. Laser drilling was performed in these blastocysts before embryo transfer. In this study, 2 blastocysts including at least one high-quality blastocyst were transplanted in each patient. In this study, according to Gardner scoring system^14^, the blastocysts with above stage 3 of blastocyst cavity expansion, above grade B of inner cell mass and above grade B of trophectoderm were regarded as high-quality blastocysts.

### Luteal phase support and pregnancy diagnosis

After blastocyst transfer, 60 mg of progesterone was intramuscularly injected once a day. Patients took progynova as that before blastocyst transfer. On the 14th and 18th days after blastocyst transfer, the blood β-hCG level > 5 mIU/ml indicated biochemical pregnancy. On the 28th day after blastocyst transfer, B-ultrasound showing a gestational sac was regarded as clinical pregnancy. For the patients with pregnancy, luteal phase support lasted for first 8–10 gestational weeks. The termination of pregnancy occurring within 28 gestational weeks was regarded as abortion.

### Statistical analysis

All data were analyzed with SPSS 22.0 software. Measurement data were expressed as mean ± standard deviation, and *t* test was used for comparison between groups. Counting data were expressed as rate, and χ^2^ test was used for comparison among groups and Fisher's exact test was used for theoretical frequency less than 5. Logistic regression equation was used for multivariate analysis, and ROC curve was used for correlation analysis between clinical pregnancy and serum E2 level. Statistical significance was established at *P* < 0.05.

## Results

### Comparisons of clinical characteristics and pregnancy outcomes among groups divided by serum estrogen levels on the endometrium transformation day

#### Comparisons of clinical characteristics among groups

According to quartile (P25) of serum estrogen levels on the endometrium transformation day, the 708 cycles were divided into group A_1_ (E2 < 157.5 pg/ml, 176 cycles), group A_2_ (157.5 pg/ml ≤ E < 206.4 pg/ml, 178 cycles), group A_3_ (206.4 pg/ml ≤ E2 < 302.3 pg/ml, 176 cycles) and group A_4_ (E2 ≥ 302.3 pg/ml, 178 cycles). The days of taking progynova was significantly different among groups (*P* < 0.05), but there were no statistical differences in the mean age, endometrial thickness both on the endometrium transformation day and on the blastocyst transfer day, progesterone level on the blastocyst transfer day and number of high-quality blastocysts transferred among groups (*P* > 0.05) (Table 1).

**Table 1:** Comparisons of clinical characteristics among groups divided by serum estrogen levels on the endometrium transformation day.

Groups	Age (years)	Endometrial thickness on the transformation day (mm)	Endometrial thickness on the blastocyst transfer day (mm)	Days of taking progynova	P level on the blastocyst transfer day (ng/ml)	Number of high-quality blastocysts transferred
A_1_ (n = 176)	30.16 ± 3.42	9.89 ± 1.43	10.66 ± 1.61	13.25 ± 1.70	15.09 ± 5.74	1.88 ± 0.41
A_2_ (n = 178)	30.10 ± 3.16	10.02 ± 1.49	10.45 ± 1.75	13.51 ± 1.85	14.85 ± 6.96	1.81 ± 0.50
A_3_ (n = 176)	30.78 ± 3.41	9.74 ± 1.44	10.45 ± 1.97	13.76 ± 2.13	14.67 ± 6.58	1.80 ± 0.48
A_4_ (n = 178)	30.33 ± 3.54	9.89 ± 1.61	10.44 ± 1.69	14.49 ± 2.10	14.12 ± 7.46	1.83 ± 0.46
*F*	1.477	1.039	0.641	13.268	0.666	3.911
*P*	0.220	0.375	0.589	0.000	0.573	0.271

#### Comparisons of pregnancy outcomes between groups

Ectopic pregnancy rate was not analyzed because only 3 patients had ectopic pregnancy. There were no statistical differences in clinical pregnancy rate, abortion rate and live birth rate among groups (*P* > 0.05). Although the clinical pregnancy rate and live birth rate were lower in the group A_4_ (E2 ≥ 302.3 pg/ml on the endometrium transformation day) than other groups, these differences were not statistically different (*P* > 0.05). The group A_4_ (E2 ≥ 302.3 pg/ml on the endometrium transformation day) was significantly lower than other groups in blastocyst implantation rate and multiple-pregnancy rate (*P* < 0.05) (Table 2).

**Table 2:** Comparison of pregnancy outcomes between groups divided by serum estrogen levels on the endometrium transformation day [% (n/n)].

Group	Clinical pregnancy rate	Implantation rate	Multiple-pregnancy rate	Abortion rate	Live birth rate
A_1_	75.6 (133/176)	62.8 (221/352)	50.0 (88/176)	9.0 (12/133)	68.8 (121/176)
A_2_	70.8 (126/178)	52.8 (188/356)	36.0 (64/178)	11.1 (14/126)	62.4 (111/178)
A_3_	77.8 (137/176)	59.4 (209/352)	40.9 (72/176)	14.6 (20/137)	66.5 (117/176)
A_4_	69.7 (124/178)	50.0* (178/356)	32.6* (58/178)	11.3 (14/124)	60.7 (108/178)
χ^2^	4.101	14.864	12.696	2.112	3.184
p	0.251	0.002	0. 005	0.550	0.364

*Compared with other groups, *P* < 0.05.

### Comparisons of clinical characteristics and pregnancy outcomes among groups divided by serum estrogen levels on the blastocyst transfer day

#### Comparisons of clinical characteristics among groups

According to quartile (P25) of serum estrogen levels on the frozen-thawed blastocyst transfer day, the 708 cycles were divided into group B_1_ (E2 < 147 pg/ml, 176 cycles), group B_2_ (147 pg/ml ≤ E2 < 200.4 pg/ml, 178 cycles), group B_3_ (200.4 pg/ml ≤ E2 < 323 pg/ml, 176 cycles) and group B_4_ (E2 ≥ 323 pg/ml, 178 cycles). The days of taking progynova was significantly different among groups (*P* < 0.05), but there were no statistical differences in the mean age, endometrial thickness on the endometrium transformation day, progesterone level and endometrial thickness on the blastocyst transfer day, and number of high-quality blastocysts transferred among groups (*P* > 0.05) (Table 3).

**Table 3:** Comparison of clinical characteristics among groups divided by serum estrogen levels on the blastocyst transfer day.

Groups	Age (years)	Endometrial thickness on the transformation day (mm)	Endometrial thickness on the blastocyst transfer day (mm)	Days of taking progynova	P level on the blastocyst transfer day (ng/ml)	Number of high-quality blastocysts transferred
B_1_ (n = 176)	30.11 ± 3.13	9.92 ± 1.48	10.59 ± 1.57	13.23 ± 1.77	15.54 ± 6.52	1.81 ± 0.50
B_2_ (n = 178)	30.39 ± 3.54	9.89 ± 1.52	10.39 ± 1.93	13.15 ± 1.60	14.74 ± 6.27	1.88 ± 0.36
B_3_ (n = 176)	30.63 ± 3.38	9.95 ± 1.51	10.47 ± 1.85	13.84 ± 1.99	14.70 ± 6.87	1.78 ± 0.54
B_4_ (n = 178)	30.24 ± 3.49	9.79 ± 1.47	10.56 ± 1.65	14.78 ± 2.18	13.74 ± 7.07	1.84 ± 0.45
*F*	0.754	0.349	0.476	27.689	2.135	2.669
*P*	0.520	0.790	0.699	0.000	0.094	0.446

#### Comparisons of pregnancy outcomes among groups

There were no statistical differences in clinical pregnancy rate, blastocyst implantation rate, multiple-pregnancy rate, abortion rate and live birth rate among groups (*P* > 0.05) (Table 4).

**Table 4:** Comparison of pregnancy outcomes between groups divided by serum estrogen levels on the blastocyst transfer day [% (n/n)].

Group	Clinical pregnancy rate	Implantation rate	Multiple-pregnancy rate	Abortion rate	Live birth rate
B_1_	76.1 (134/176)	58.8 (207/352)	41.5 (73/176)	7.0 (12/134)	69.3 (122/176)
B_2_	70.2 (125/178)	53.4 (190/356)	37.1 (66/178)	8.1 (17/125)	60.7 (108/178)
B_3_	75.0 (132/176)	57.1 (201/352)	40.9 (72/176)	11.8 (18/132)	63.6 (112/176)
B_4_	72.5 (129/178)	55.6 (198/356)	39.9 (71/178)	4.5 (13/129)	64.6 (115/178)
χ^2^	1.905	2.295	0.847	1.900	2.982
*P*	0.592	0.514	0.838	0.593	0.394

### Comparisons of clinical characteristics and hormone levels between pregnant group and non-pregnant group

According to different clinical outcomes, the 708 cycles were divided into clinical pregnant group (n = 520) and non-pregnant group (n = 188). The mean age was significantly younger and the number of high-quality blastocysts transferred was significantly higher in the clinical pregnant group than in the non-clinical pregnant group (*P* < 0.05), but endometrial thicknesses on both endometrium transformation day and blastocyst transfer day, days of taking progynova and progesterone level on the blastocyst transfer day were not significantly different between the two groups (*P* > 0.05). Although E2 levels on both endometrium transformation day and blastocyst transfer day were all lower in the pregnant group than in the non-pregnant group, these differences were not statistically significant (*P* > 0.05) (Table 5).

**Table 5:** Comparisons of clinical characteristics and hormone levels between pregnant group and non-pregnant group.

Groups	Age (year)	Endometrial thickness on the transformation day (mm)	Endometrial thickness on the embryo transfer day (mm)	Days of taking progynova	E2 level on the transformation day (pg/ml)	E2 level on the embryo transfer day (pg/ml)	P level on the embryo transfer day (ng/ml)	Number of high-quality blastocysts transferred
Clinical pregnant group	30.09 ± 3.29	9.943 ± 1.56	10.54 ± 1.73	13.80 ± 2.07	328.07 ± 398.31	341.53 ± 376.97	13.56 ± 6.68	1.87 ± 0.52
Non-pregnant group	31.03 ± 3.56	9.730 ± 1.27	10.38 ± 1.81	13.61 ± 1.81	393.94 ± 453.49	389.87 ± 460.04	14.38 ± 6.78	1.73 ± 0.43
*F*	2.668	0.942	0.554	1.306	1.036	0.743	0.554	10.423
*P*	0.008	0.346	0.579	0.192	0.300	0.458	0.579	0.001

### Multivariate regression analysis for clinical pregnancy

Multivariate regression analysis indicated that the age and number of high-quality blastocysts transferred were independent factor affecting clinical pregnancy (*P* < 0.05). Nevertheless, endometrial thickness, estrogen level, days of taking progynova and progesterone level on the blastocyst transfer day failed to significantly affect clinical pregnancy (*P* > 0.05) (Table 6).

**Table 6:** Multivariate regression analysis for clinical pregnancy.

Factors	OR	95% CI	*P*
Age	0.927	0.882–0.974	0.003
Endometrial thickness on the transformation day	1.088	0.955–1.240	0.203
Endometrial thickness on the blastocyst transfer day	1.021	0.917–1.137	0.705
Days of taking progynova	1.086	0.988–1.194	0.086
Estrogen level on the transformation day	1.000	0.999–1.000	0.275
Estrogen level on the blastocyst transfer day	1.000	0.999–1.000	0.246
Progesterone level on the blastocyst transfer day	1.016	0.990–1.044	0.232
Number of high-quality blastocysts transferred	1.723	1.231–2.411	0.002

### Correlation analysis between clinical pregnancy and serum E2 level

**Figure 1:**
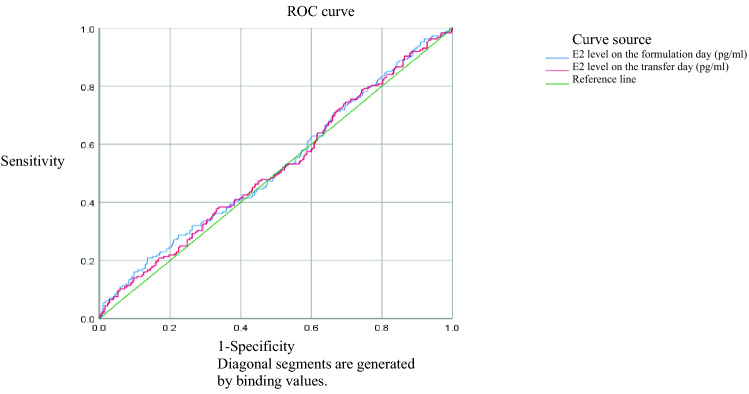
Correlation analysis between clinical pregnancy and serum E2 level.

A ROC curve was drawn using the serum E2 levels of non-clinical pregnancy group on the transformation day and blastocyst transfer day as variables. The area under the curve of serum E2 on the transformation day was 0.525 (*P* = 0.300), and the area under the curve of serum E2 on the blastocyst transfer day was 0.518 (*P* = 0.458), suggesting that the serum estrogen levels on the transformation day and the blastocyst transfer day were not significantly correlated with clinical pregnancy (*P* > 0.05) (Fig. 1).

## Discussion

It is not clear that in HRT-FET cycles, what level the serum estrogen should be maintained at. There are different reports about the effects of E2 level on pregnancy outcome in HRT-FET cycles^8–11^. High serum estradiol level might lead to gland stroma asynchronization, interfering with endometrial receptivity and affecting pregnancy outcomes^15^. The E2 level in the proliferative phase was negatively correlated with ongoing pregnancy rate and live birth rate^16^. In the patients with E2 higher than normal level, the live birth rate increased after FET^17^. Above different results may be due to different exogenous estrogen supplementation protocols or different serum E2 assessment standards^10^. This study indicated that in HRT-FET cycles, as long as the endometrial thickness reached 8 mm or more, the estrogen levels on both endometrium transformation day and blastocyst transfer day did not affect pregnancy outcomes. Therefore, we believe that the serum estrogen level before HRT-FET is not significantly associated with pregnancy outcome, which is consistent with other reports^8–11^. It has been reported that the high serum E2 level on the endometrium transformation day affects embryo implantation, pregnancy rate, ongoing pregnancy rate and live birth rate for cleavage embryos, but has no adverse effects on frozen blastocyst transfer^18^. This study indicated that the group A_4_ (E2 ≥ 302.3 pg/ml on endometrium transformation day) was significantly lower than other groups in embryo implantation rate (50%) and multiple-pregnancy rate (32.6%) (*P* < 0.05). This result suggests that the high estrogen level on the endometrium transformation day may affect embryo implantation, thus effectively reducing multiple-pregnancy rate. It also has been reported that low progesterone level on the day of embryo transfer (< 8.8 ng/ml) affects pregnancy outcomes^19^. In this study, the mean progesterone level on the day of blastocyst transfer was 10 ng/ml, no less than 8.8 ng/ml of progesterone was detected in these patients on the day of blastocyst transfer. Multivariate regression analysis of this study indicated that the age and number of high-quality blastocysts transferred were independent factor affecting clinical pregnancy, while the estrogen levels before blastocyst transfer, days of taking progynova and progesterone level on the blastocyst transfer day failed to significantly affect clinical pregnancy. Drawing ROC curves using the serum E2 levels of non-clinical pregnancy group on the transformation day and blastocyst transfer day as variables, obtained the area (0.525, *P* = 0.300) under the curve of serum E2 on the transformation day and the area (0.518, *P* = 0.458) under the curve of serum E2 on the embryo transfer day. This suggests that the serum estrogen levels on the transformation day and the blastocyst transfer day were not significantly correlated with clinical pregnancy. In this study, preparation for blastocyst transfer began when the endometrial thickness reached more than 8 mm, so it is not possible to show the effects of endometrial thickness on pregnancy outcome.

In HRT-FET cycles, the role of estrogen may depend on the endometrial thickness. The estrogen binds with estrogen receptors in the endometrium to secrete vascular endothelial growth factor, basic fibroblast growth factor and transforming growth factor β 1, and these factors promote endometrial growth^20^. In some patients, activating estrogen receptor and its downstream signaling pathway may require high E2 levels before embryo transfer to promote endometrial growth. However, the effect of the serum E2 level markedly higher than physiological level, on the endometrium did not show a sustained dose-dependent growth^8^. In this study, patients took progynova. In order to reach endometrial thickness of 8 mm or more, the dosage and days of taking progynova were adjusted for different patients, so the days of taking progynova were significantly different among groups. This suggests that the endometrium only respond to high-level and long-time estrogen stimulation in some patients. In this study, there was no excessive endometrial hyperplasia, so the endometrial thicknesses on both endometrium transformation day and blastocyst transfer day were not significantly different among groups. This study suggests that endometrium transformation may begin when the endometrial thickness reaches standard level without excessive focus on estrogen levels and days of taking progynova in HRT-FET cycles.

In summary, the serum E2 levels before blastocyst transfer do not affect clinical pregnancy rate and live birth rate in the HRT-FET cycles. In clinical practice, endometrium transformation may begin when the endometrial thickness reaches standard level without excessive focus on estrogen levels and days of taking progynova in HRT-FET cycles.

## Data Availability

The datasets used and/or analyzed during the current study available from the corresponding author on reasonable request.

## References

[1] Groenewoud ER, Cohlen BJ, Macklon NS (2018). Programming the endometrium for deferred transfer of cryopreserved embryos: Hormone replacement versus modified natural cycles. Fertil. Steril..

[2] Fei H, Jia L, Yingying Z (2018). Application value of three endometrial preparation schemes in frozen-thawed embryo transfer. Shenzhen J. Integr. Tradit. Chin. West. Med..

[3] Jie Y, Wenling Z, Xinru G (2020). Outcome analysis of two intimal preparation schemes in freeze-thaw embryo transfer. China Health Stand. Manag..

[4] Shaodi Z, Qiuyuan L, Yisha Y, Cuilian Z (2020). The effect of endometrial thickness on pregnancy outcomes of frozen-thawed embryo transfer cycles which underwent hormone replacement therapy. PLoS One.

[5] Xu H, Zhang XQ, Zhu XL, Weng HN, Xu LQ, Huang L, Liu FH (2021). Comparison of vaginal progesterone gel combined with oral dydrogesterone versus intramuscular progesterone for luteal support in hormone replacement therapy-frozen embryo transfer cycle. J. Gynecol. Obstet. Hum. Reprod..

[6] Haiyan L, Gang Y, Yu L, Lin L, Xiaoli C, Qingxue Z (2022). Does serum progesterone level impact the ongoing pregnancy rate in frozen embryo transfer under artificial preparation with vaginal progesterone? Study protocol for a randomized controlled trial. Trials.

[7] Reed BG, Carr BR, Feingold KR, Anawalt B, Boyce A (2018). The normal menstrual cycle and the control of ovulation. Endotext.

[8] Ling D, Xin C, Desheng Y, Shiling C (2018). Effect of serum estradiol level before progesterone administration on pregnancy outcomes of frozen-thawed embryo transfer cycles. J. South. Med. Univ..

[9] Celik C, Asoglu MR, Karakis LS, Findikli N, Gultomruk M, Cavkaytar S, Bahceci M (2019). The impact of serum oestradiol concentration prior to progesterone administration on live birth rate in single vitrified-warmed blastocyst transfer cycles. Reprod. Biomed. Online.

[10] Mackens S, Santos-Ribeiro S, Orinx E, De Munck N, Racca A, Roelens C (2020). Impact of serum estradiol levels prior to progesterone administration in artificially prepared frozen embryo transfer cycles. Front. Endocrinol. (Lausanne).

[11] Palmerola KL, Rudick BJ, Lobo RA (2018). Low estradiol responses in oocyte donors undergoing gonadotropin stimulation do not influence clinical outcomes. J. Assist. Reprod. Genet..

[12] Labarta E, Mariani G, Holtmann N, Celada P, Remohí J, Bosch E (2017). Low serum progesterone on the day of embryo transfer is associated with a diminished ongoing pregnancy rate in oocyte donation cycles after artificial endometrial preparation: A prospective study. Hum. Reprod..

[13] Gaggiotti-Marre S, Martinez F, Coll L, Garcia S, Álvarez M, Parriego M (2019). Low serum progesterone the day prior to frozen embryo transfer of euploid embryos is associated with significant reduction in live birth rates. Gynecol. Endocrinol..

[14] Gardner DK, Lane M, Stevens J, Schlenker T, Schoolcraft WB (2000). Blastocyst score affects implantation and pregnancy outcome: Toward a single blastocyst transfer. Fertil. Steril..

[15] Şanverdi İ, Özkaya E, Kutlu T, Şenol T, Akalın M, SayarAkalın E, Şahin Y, Karateke A (2016). Non-invasive prediction of implantation window in controlled hyperstimulation cycles: Can the time from the menstrual day at embryo transfer to expected menstrual cycle give a clue?. Turk. J. Obstet. Gynecol..

[16] Fritz R, Jindal S, Feil H, Buyuk E (2017). Elevated serum estradiol levels in artificial autologous frozen embryo transfer cycles negatively impact ongoing pregnancy and live birth rates. J. Assist. Reprod. Genet..

[17] Sarkar P, Gandhi A, Plosker S, Ying Y, Mayer J, Imudia AN (2018). Does supraphysiologic estradiol level during IVF have any effect on oocyte/embryo quality? A sibling embryo cohort analysis of fresh and subsequent frozen embryo transfer. Minerva Ginecol..

[18] Li Q, Ruan L, Zhu L, Yang Z, Zhu M, Luo Y (2022). Elevated estradiol levels in frozen embryo transfer have different effects on pregnancy outcomes depending on the stage of transferred embryos. Sci. Rep..

[19] Labarta E, Mariani G, Paolelli S, Rodriguez-Varela C, Vidal C, Giles J, Bellver J, Cruz F, Marzal A, Celada P, Olmo I, Alamá P, Remohi J, Bosch E (2021). Impact of low serum progesterone levels on the day of embryo transfer on pregnancy outcome: A prospective cohort study in artificial cycles with vaginal progesterone. Hum. Reprod..

[20] Johary J, Xue M, Zhu X, Xu D, Velu PP (2013). Efficacy of estrogen therapy in patients with intrauterine adhesions: Systematic review. J. Minim. Invasive Gynecol..

